# Military Surgery

**Published:** 1916-10

**Authors:** 


### ABSTRACTS

Have you ever felt that this department of the BUFFALO MEDICAL JOURNAL was lacking in abstracts of your specialty, or that such as were published lacked the expert judgement which you yourself could have exercised in selection and preparation? If not, you are more charitable than the editor has been to himself. If so, will you assist in abstracting from a few journals, and thus make this department more representative and more valuable? Incidentally, there are few forms of study more helpful to the worker than this.

### Military Surgery.

**Autoplasty of the Corpora Cavernosa With Fascia Lata.** F. Legeue, Soc. de Chir., Nov. 3, 1915, reports a civil case in which a large fibro-osseous plaque was removed from the sheath, leaving too large an aperture in the sheath for direct suture. A square was cut from the fascia lata, sewed in place, the immediate and remote results (after 9 months) being good.

